# Neutrophil to lymphocyte ratio, not platelet to lymphocyte or lymphocyte to monocyte ratio, is predictive of patient survival after resection of early-stage pancreatic ductal adenocarcinoma

**DOI:** 10.1186/s12885-020-07182-9

**Published:** 2020-08-11

**Authors:** David T. Pointer, David Roife, Benjamin D. Powers, Gilbert Murimwa, Sameh Elessawy, Zachary J. Thompson, Michael J. Schell, Pamela J. Hodul, Jose M. Pimiento, Jason B. Fleming, Mokenge P. Malafa

**Affiliations:** 1 Department of Gastrointestinal Oncology, H. Lee Moffitt Cancer Center and Research Institute, 12902 USF Magnolia Dr, Tampa, FL 33612 USA; 2 Department of Surgery, University of Texas Southwestern, Dallas, TX USA; 3 Department of Biostatistics and Bioinformatics, H. Lee Moffitt Cancer Center and Research Institute, Tampa, FL USA

**Keywords:** Neutrophil lymphocyte ratio, Platelet lymphocyte ratio, Lymphocyte monocyte ratio, Pancreatic cancer, Biomarker

## Abstract

**Background:**

NLR, PLR, and LMR have been associated with pancreatic ductal adenocarcinoma (PDAC) survival. Prognostic value and optimal cutpoints were evaluated to identify underlying significance in surgical PDAC patients.

**Methods:**

NLR, PLR, and LMR preoperative values were available for 277 PDAC patients who underwent resection between 2007 and 2015. OS, RFS, and survival probability estimates were calculated by univariate, multivariable, and Kaplan-Meier analyses. Continuous and dichotomized ratio analysis determined best-fit cutpoints and assessed ratio components to determine primary drivers.

**Results:**

Elevated NLR and PLR and decreased LMR represented 14%, 50%, and 50% of the cohort, respectively. OS (*P* = .002) and RFS (*P* = .003) were significantly decreased in resected PDAC patients with NLR ≥5 compared to those with NLR < 5. Optimal prognostic OS and RFS cutpoints for NLR, PLR, and LMR were 4.8, 192.6, and 1.7, respectively. Lymphocytes alone were the primary prognostic driver of NLR, demonstrating identical survival to NLR.

**Conclusions:**

NLR is a significant predictor of OS and RFS, with lymphocytes alone as its primary driver; we identified optimal cutpoints that may direct future investigation of their prognostic value. This study contributes to the growing evidence of immune system influence on outcomes in early-stage pancreatic cancer.

## Background

Pancreatic ductal adenocarcinoma (PDAC) is the third leading cause of cancer-related death in the US, with an estimated 45,750 deaths in 2019 and a 5-year overall survival (OS) rate of 9% [1]. Among newly diagnosed PDAC patients, only 15 to 20% present with resectable disease. With resection as the only chance for cure, prognosis is generally poor with reported 5-year OS of 10–30% after resection [2–6]. AJCC TNM staging is the only widely accepted indicator of prognosis for resectable pancreatic cancer; however its performance in early-stage disease has been questioned [7]. Additionally, controversy regarding initial treatment of early-stage pancreatic cancer persists, yielding no uniform treatment algorithm. Given the wide variation in the biological behavior of PDAC and treatment algorithms for this disease, there is an unmet need for enhanced prognostic biomarkers. Biomarkers derived from easily obtainable laboratory values have shown potential to meet this need and may help to stratify patients with early-stage pancreatic cancer and guide future treatment plans.

Conventionally, survival outcomes among cancer patients have been determined by the disease stage and receipt of treatment. More recently, however, increased attention has been directed toward the role of inflammation and immune response in the tumor microenvironment and their effects on tumor behavior. Quantifying the systemic inflammatory response by C-reactive protein and various nutritional parameters has shown prognostic significance in gastrointestinal, gynecological and thoracic cancers [8]. Additionally, inflammatory indices and immunologic ratios, including ratios comprised of intratumoral or circulating neutrophils, platelets, lymphocytes, and monocyte counts, have been proposed to be prognostic biomarkers for a wide range of malignancies [9–12].

The neutrophil to lymphocyte ratio (NLR), platelet to lymphocyte ratio (PLR), and lymphocyte to monocyte ratio (LMR) are among the many surrogate biomarkers for inflammation that have been associated with outcomes in gastrointestinal cancers. Although these ratios have been reported to have promising prognostic value, few studies have examined the effect of these inflammatory ratios in US surgical cohorts [12–18]. Moreover, many single-institution studies have reported inconsistent prognostic outcomes for these surrogate biomarkers. We previously reported an inverse association between survival and NLR in patients with borderline resectable disease [14]. To expand the scope of our previous analysis, we evaluated the prognostic significance of the NLR, PLR, and LMR in a cohort of patients with resected PDAC who were treated at a high-volume cancer center. Furthermore, we aimed to establish optimal NLR, PLR, and LMR cutpoints for determining OS and recurrence-free survival (RFS) and define the primary factor driving the prognostic value of these ratios for survival outcomes. We hypothesized that preoperatively increased NLR and PLR and decreased LMR were associated with worse OS in patients with resectable PDAC.

## Methods

A retrospective review was conducted using our institutional prospective pancreatic cancer database, as part of our ongoing outcome-based study. The study was approved by our Institutional Review Board (MCC#16446), and patient consent was unable to be obtained as this study was conducted retrospectively on de-identified data, posing less than minimal risk. Patients diagnosed with PDAC who underwent curative-intent resection for the treatment of their disease were identified. Resectable and borderline resectable PDAC patients were defined and included on the basis of the NCCN guidelines applied at the time of diagnosis. Pancreatic resection included open or minimally invasive pancreaticoduodenectomy, total pancreatectomy, and distal pancreatectomy performed at our institution.

Patient characteristics were summarized using descriptive statistics, including median and range for continuous measures and proportions and frequencies for categorical measures. Kaplan-Meier plots were made to determine OS and RFS for the NLR, PLR, and LMR. Survival probability estimates were calculated using the Kaplan-Meier method. Univariate and multivariable Cox proportional-hazard models for OS and RFS were run for each ratio as continuous predictors and dichotomized forms. The NLR, PLR, and LMR were calculated by dividing the absolute neutrophil count by the lymphocyte count, the platelet count by the lymphocyte count, and the lymphocyte count by the monocyte count, respectively. Dichotomized analyses included neutrophil and lymphocyte counts and percentages, which were defined as the proportion of neutrophils or lymphocytes to all white blood cells in the sample. Values used for these calculations were part of the last complete blood count and differential obtained after neoadjuvant therapy and before operative intervention. Cutpoints of 5, 144.4, and 2.9 were used for NLR, PLR, and LMR, respectively. NLR cutpoints were determined on the basis of values used in previously published studies [15, 19]. Cutpoints for PLR and LMR were not well established; therefore, the medians of the observed data were used.

Optimal NLR, PLR, and LMR cutpoints for the prediction of OS and RFS were determined using maximally selected rank statistics based on the log-rank method [20]. The resulting cutpoint for each ratio provided the best separation of the responses into 2 groups (in which the standardized rank statistics take their maximum). The *P* value approximation was based on the improved Bonferroni inequality [21]. Variables were evaluated in relation to OS and RFS for predetermined cutpoints and newly identified best-fit cutpoints. All analyses were performed using R software (version 3.6.1).

## Results

A total of 307 patients treated at our institution between 2007 and 2015 were eligible for this study. Two hundred seventy-seven patients with complete data met the inclusion criteria and were included in the analysis. The mean age was 68 (±10) years, 57% of whom were male. Twenty-five percent of patients had a Charlson Comorbidity Index (CCI) ≤ 3, 49% had a CCI of 4 to 5, and 26% had a CCI ≥ 6. Medicare with a private supplement was the largest represented insurance provider among patients (42%). Sixty-four percent of our cohort was classified as resectable and treated with upfront resection, and 37% received neoadjuvant systemic therapy. Margin negative (R0) resection was achieved in 90% of our patients, with 70 and 82% demonstrating lymphovascular and perineural invasion, respectively (Table 1).

**Table 1:** Descriptive statistics of study cohorts

Demographics	Overall ***N*** = 277	NLR < 5 ***N*** = 239	NLR ≥ 5 ***N*** = 38	***P*** Value	PLR < 144.4 ***N*** = 139	PLR ≥ 144.4 ***N*** = 138	***P*** Value	LMR ≤ 2.9 ***N*** = 140	LMR > 2.9 ***N*** = 137	***P*** Value
**Age, median (range), y**	68.0 (33.0–90.0)	68.0 (33.0–90.0)	67.5 (47.0–86.0)	.396	69.0 (40.0–90.0)	66.5 (33.0–86.0)	.384	68.0 (33.0–86)	67.0 (40.0–90.0)	.083
**Sex**, no. (%)				.715			.103			.239
Female	120 (43.3)	102 (42.7)	18 (47.4)		53 (38.1)	67 (48.6)		66 (47.1)	54 (39.4)	
Male	157 (56.7)	137 (57.3)	20 (52.6)		86 (61.9)	71 (51.4)		74 (52.9)	83 (60.6)	
**Race**, no. (%)				.166			.279			.383
Black	11 (3.97)	11 (4.60)	0 (0.00)		3 (2.16)	8 (5.80)		4 (2.86)	7 (5.11)	
Other	11 (3.97)	8 (3.35%)	3 (7.89)		5 (3.60)	6 (4.35)		4 (2.86)	7 (5.11)	
White	255 (92.1)	220 (92.1%)	35 (92.1)		131 (94.2)	124 (89.9)		132 (94.3)	123 (89.8)	
**BMI, median (range)**	26.3 (16.7–58.5)	26.4 (16.7–58.5)	26.2 (18.6–44.1)	.841	26.3 (16.7–46.7)	26.3 (16.7–58.5)	.976	26.4 (16.7–55.7)	26.2 (16.7–58.5)	.848
**CCI**, no. (%)				.646			.900			.157
0–3	69 (24.9)	58 (24.3)	11 (28.9)		33 (23.7)	36 (26.1)		28 (20.0)	41 (29.9)	
4–5	136 (49.1)	120 (50.2)	16 (42.1)		69 (49.6)	67 (48.6)		74 (52.9)	62 (45.3)	
≥ 6	72 (26.0)	61 (25.5)	11 (28.9)		37 (26.6)	35 (25.4)		38 (27.1)	34 (24.8)	
**Tumor Size**	2.70 (0.08–8.20)	2.70 (0.08–8.20)	2.70 (0.60–6.50)	.593	3.00 (0.10–8.20)	2.5 (0.08–7.00)	.028	2.60 (0.08–7.00)	2.90 (0.10–8.20)	.123
**Pathologic Stage**, no. (%)				.439			.126			.642
T0	18 (6.55)	17 (7.17)	1 (2.63%)		9 (6.52)	9 (6.57)		11 (7.91)	7 (5.15)	
T1, no. (%)	23 (8.36)	18 (7.59)	5 (13.2)		16 (11.6)	7 (5.11)		10 (7.19)	13 (9.56)	
T2, no. (%)	132 (48.0)	116 (48.9)	16 (42.1)		69 (50.0)	63 (46.0)		69 (49.6)	63 (46.3)	
T3	102 (37.1)	86 (36.3)	16 (42.1)		44 (31.9)	58 (42.3)		49 (35.3)	53 (39.0)	
**Preoperative Resectability**, no. (%)				.186			.005			<.001
Borderline	101 (36.5)	83 (34.7)	18 (47.4)		39 (28.1)	62 (44.9)		68 (48.6)	33 (24.1)	
Resectable	176 (63.5)	156 (65.3)	20 (52.6)		100 (71.9)	76 (55.1)		72 (51.4)	104 (75.9)	
**Neoadjuvant Therapy**, no. (%)				.204			.008			<.001
No	175 (63.2)	155 (64.9)	20 (52.6)		99 (71.2)	76 (55.1)		71 (50.7)	104 (75.9)	
Yes	102 (36.8)	84 (35.1)	18 (47.4)		40 (28.8)	62 (44.9)		69 (49.3)	33 (24.1)	
**Margin**, no. (%)				1.00			1.00			.145
Negative	249 (89.9)	215 (90.0)	34 (89.5)		125 (89.9)	124 (89.9)		130 (92.9)	119 (86.9)	
Positive	28 (10.1)	24 (10.0)	4 (10.5)		14 (10.1)	14 (10.1)		10 (7.14)	18 (13.1)	
**Lymphovascular Invasion**, no. (%)				1.00			.855			.188
No	83 (30.5)	72 (30.5)	11 (30.6)		43 (31.4)	40 (29.6)		47 (34.6)	36 (26.5)	
Yes	189 (69.5)	164 (69.5)	25 (69.4)		94 (68.6)	95 (70.4)		89 (65.4)	100 (73.5)	
**Perineural Invasion**, no. (%)				.606			.680			.159
No	50 (18.4)	45 (19.1)	5 (13.9)		27 (19.7)	23 (17.0)		30 (22.1)	20 (14.7)	
Yes	222 (81.6)	191 (80.9)	31 (86.1)		110 (80.3)	112 (83.0)		106 (77.9)	116 (85.3)	
**Complication 3-4**^a^, no. (%)				.604			.359			1.00
No	241 (87.0)	209 (87.4)	32 (84.2)		124 (89.2)	117 (84.8)		122 (87.1)	119 (86.9)	
Yes	36 (13.0)	30 (12.6)	6 (15.8)		14 (10.8)	21 (15.2)		18 (12.9)	18 (13.1)	
**Completion of Adjuvant Therapy**, no. (%)				.155			1.00			.166
No	97 (35.8)	79 (33.9)	18 (47.4)		48 (35.6)	49 (36.0)		55 (40.1)	42 (31.3)	
Yes	174 (64.2)	154 (66.1)	20 (52.6)		87 (64.4)	87 (64.0)		82 (59.9)	92 (68.7)	

^a^Clavien-Dindo Classification of Surgical Complications

*Abbreviations*: *BMI* body mass index, *NLR* neutrophil to lymphocyte ratio, *PLR* platelet to lymphocyte ratio, *LMR* lymphocyte to monocyte ratio, *CCI* Charleson Comorbidity Index

**Fig. 1:**
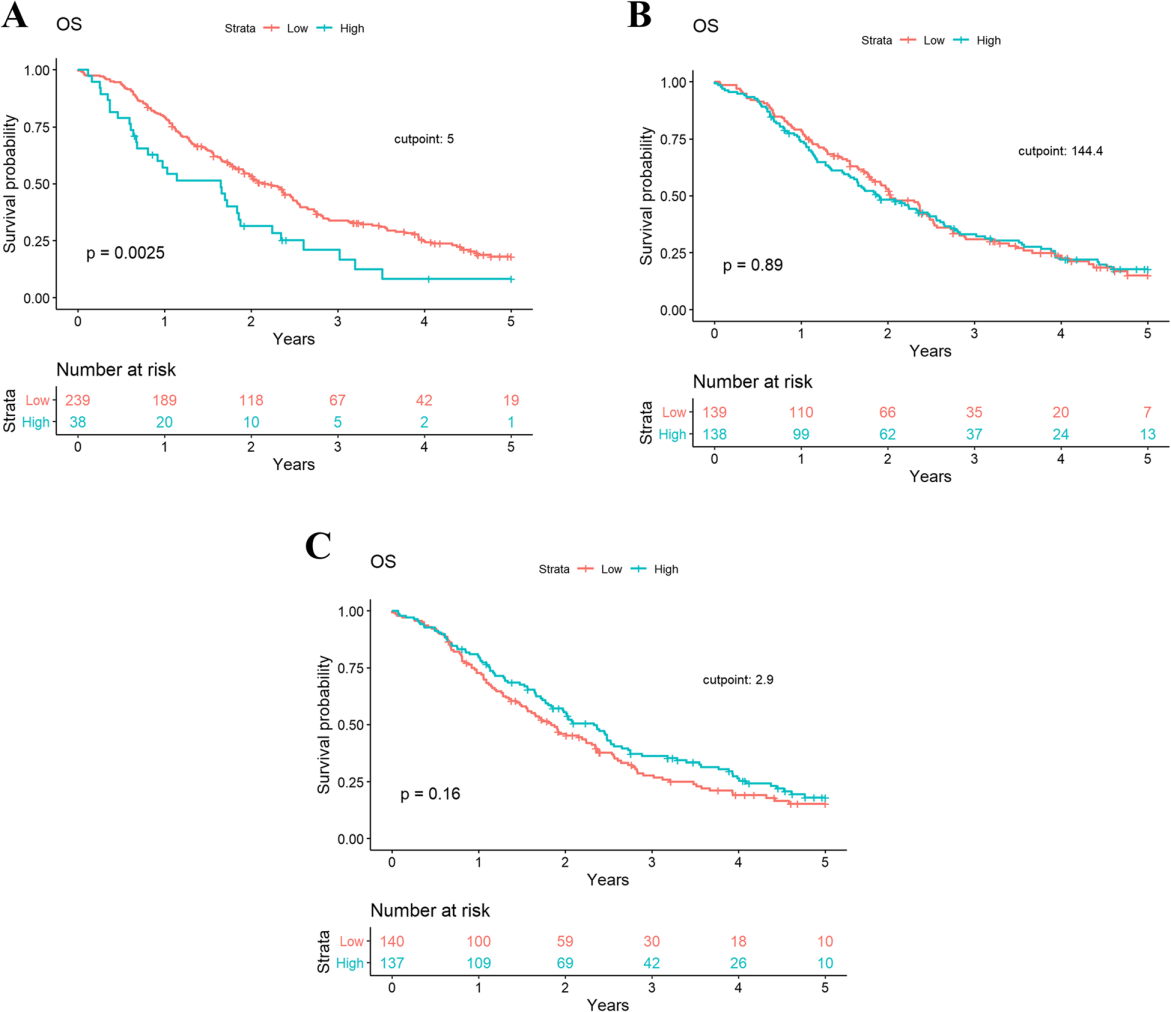
Kaplan-Meier plot demonstrating overall survival in (**a**) NLR, (**b**) PLR, and (**c**) LMR

**Fig. 2:**
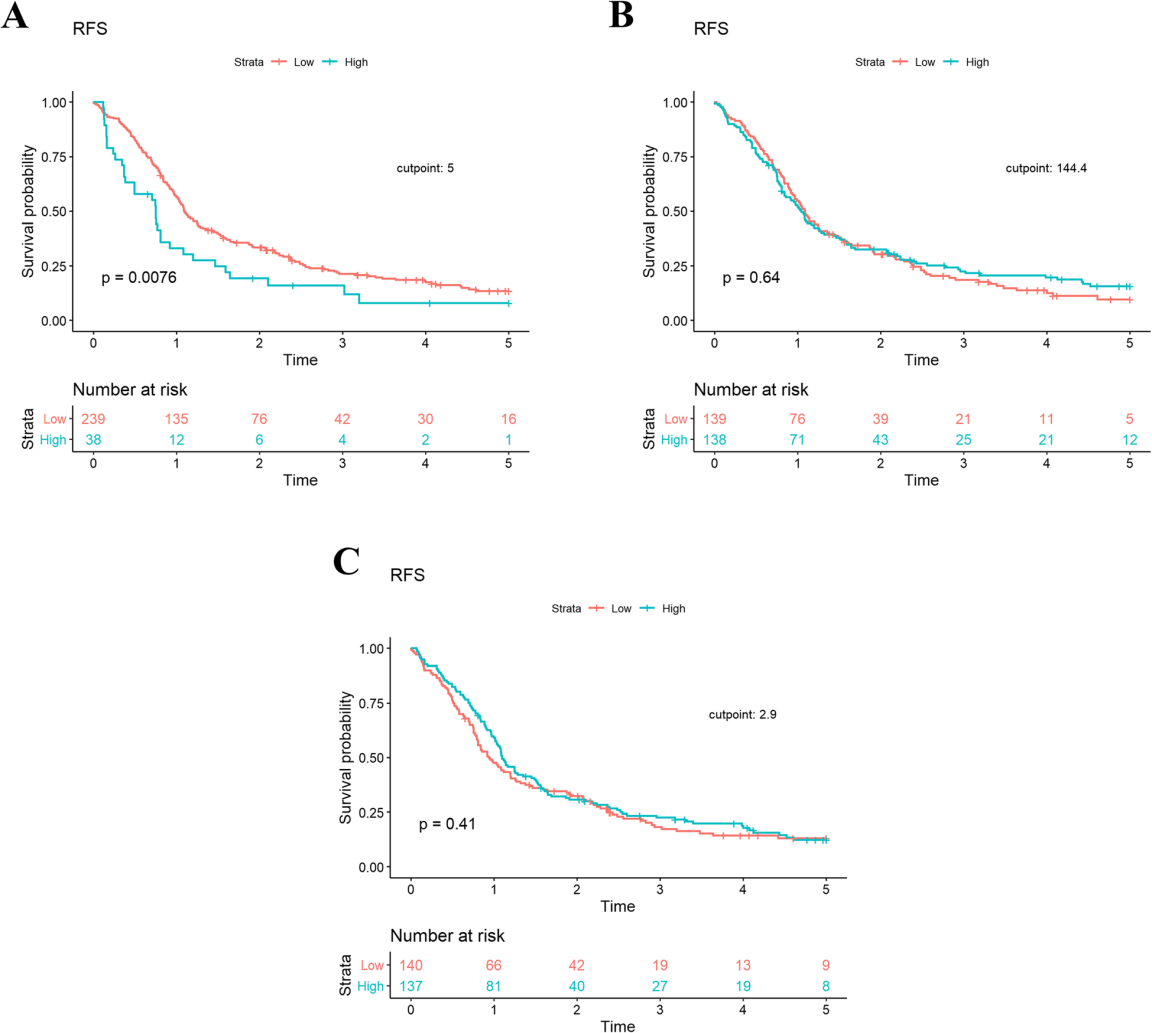
Kaplan-Meier plot demonstrating recurrence-free survival in (**a**) NLR, (**b**) PLR and (**c**) LMR

Mean preoperative NLR, PLR and LMR was 3.4 ± 2.2, 169.1 ± 95.7, and 3.1 ± 1.9, respectively (Additional File 1). Using the predetermined cutpoints described above, 14%, 50%, and 50% of patients demonstrated preoperative NLR ≥ 5, PLR ≥ 144.4, and LMR ≤ 2.9, respectively. OS was significantly shorter among patients with an NLR ≥ 5 than patients with an NLR < 5 in univariate (HR, 1.80 [95% CI, 1.22–2.64]; *P* = .003) and multivariable (HR, 2.13 [95% CI, 1.41–3.22]; *P* = .002) analyses (Table 2). Neither the PLR nor LMR demonstrated a significant association with OS (Table 2 and Fig. 1). Patients with a high NLR also demonstrated significantly worse RFS in univariate (HR, 1.65 [95% CI, 1.14–2.39]; *P* = .008) and multivariable (HR, 2.20 [95% CI, 1.43–3.39]; *P* = 0.0003) analyses (Table 3 and Fig. 2). This was not observed with PLR or LMR. In multivariable analyses, pathologic T stage, presence of grade 3/4 complications, CCI ≥ 4, NLR, node positivity, and perineural invasion were found to be significant predictors of OS and RFS (Tables 2 and 3).

**Table 2:** Univariate and multivariate Cox proportional hazard models for overall survival

Variable	Univariate Analysis HR (95% CI)	***P*** value	Multivariable Analysis HR (95% CI)^a^	***P*** value
**Gender**				
Female	N/A	N/A	1.0 (Reference)	
Male	N/A	N/A	1.33 (1.00–1.78)	.053
**Age**				
≤ 70	N/A	N/A	1.0 (Reference)	
> 70	N/A	N/A	1.41 (1.01–1.97)	.04
**Pathologic Stage**				
T0	N/A	N/A	1.0 (Reference)	
T1	N/A	N/A	1.36 (0.53–3.54)	.53
T2	N/A	N/A	2.85 (1.33–6.12)	.007
T3	N/A	N/A	3.13 (1.44–6.83)	.004
**CCI**				
0–3	N/A	N/A	1.0 (Reference)	
4+	N/A	N/A	1.63 (1.11–2.40)	.01
**NLR**				
< 5	1.0 (Reference)		1.0 (Reference)	
≥ 5	1.80 (1.22–2.64)	.003	2.13 (1.41–3.22)	.002
**PLR**				
< 144.4	1.0 (Reference)		N/A	N/A
≥ 144.4	1.02 (0.78–1.34)	.889	N/A	N/A
**LMR**				
< 2.9	1.0 (Reference)		N/A	N/A
≥ 2.9	0.82 (0.63–1.08)	.156	N/A	N/A
**Perineural Invasion**				
No	N/A	N/A	1.0 (Reference)	
Yes	N/A	N/A	1.51 (0.99–2.31)	.055
**Complication Grade (3–4)**^b^	N/A	N/A	2.37 (1.57–3.57)	<.0001
**Positive Nodes**	N/A	N/A	1.07 (1.03–1.12)	.002

^a^model includes: age, gender, pathologic stage, CCI, complication score, NLR, nodal and perineural invasion status. ^b^ Clavien-Dindo Classification of Surgical Complications

*Abbreviations*: *CCI* Charlson Comorbidity Score, *NLR* neutrophil to lymphocyte ratio, *PLR* platelet to lymphocyte ratio, *LMR* lymphocyte to monocyte ratio

**Table 3:** Univariate and multivariate Cox proportional-hazard models for recurrence-free survival

Variable	Univariate Analysis HR (95% CI)	***P*** value	Multivariable Analysis HR (95% CI)^a^	***P*** value
**Gender**				
Female	N/A		1.0 (Reference)	
Male	N/A	N/A	1.25 (0.95–1.65)	.11
**Pathologic Stage**				
T0	N/A		1.0 (Reference)	
T1	N/A	N/A	1.29 (0.55–3.00)	.56
T2	N/A	N/A	2.41 (1.21–4.80)	.01
T3	N/A	N/A	2.60 (1.27–5.29)	.009
**CCI**				
0–3	N/A		1.0 (Reference)	
4+	N/A	N/A	1.48 (1.05–2.09)	.02
**NLR**				
< 5	1.0 (Reference)		1.0 (Reference)	
≥ 5	1.65 (1.14–2.39)	.008	2.20 (1.43–3.39)	.0003
**PLR**				
< 144.4	1.0 (Reference)		N/A	
≥ 144.4	0.94 (0.73–1.22)	.64	N/A	N/A
**LMR**				
< 2.9	1.0 (Reference)		N/A	
≥ 2.9	0.90 (0.69–1.16)	.41	N/A	N/A
**Perineural Invasion**				
No	N/A		1.0 (Reference)	
Yes	N/A	N/A	1.61 (1.08–2.41)	.02
**Complication Grade (3–4)**^b^	N/A	N/A	1.64 (1.10–2.44)	.01
**Positive Nodes**	N/A	N/A	1.08 (1.03–1.12)	.0003

*Abbreviations*: *CCI* Charlson Comorbidity Score, *NLR* neutrophil to lymphocyte ratio, *PLR* platelet to lymphocyte ratio, *LMR* lymphocyte to monocyte ratio

^a^ model includes: age, gender, pathologic stage, CCI, complication score, NLR, nodal and perineural invasion status

^b^ Clavien-Dindo Classification of Surgical Complications

Maximally selected rank analyses of NLR, PLR and LMR were performed to identify optimal cutpoints for predicting OS and RFS. OS optimal cutpoints for NLR, PLR, and LMR were 4.8, 192.6, and 1.7, respectively. For RFS, cutpoints were 4.9, 120.4, and 1.7, respectively. Because neutrophil percentage is highly correlated with NLR we found the corresponding cutpoint for determining a high neutrophil percentage to be 78% (resulting in 38 patients being above the cutpoint). Similarly, lymphocyte percentage was highly negatively correlated, with NLR with a corresponding cutpoint percentage of 15%. The components of NLR was analyzed separately to evaluate their prognostic importance. The lymphocyte percentage alone yielded a survival curve that was identical to that of the NLR, whereas the neutrophil percentage KM plot was not statistically significant (Additional File 2).

## Discussion

We demonstrated a statistically significant association between preoperative NLR and both OS and RFS in PDAC patients who underwent curative-intent resection at a high-volume cancer center. PLR and LMR failed to demonstrate any correlation with survival. In addition, we identified optimal cutpoints for immunologic ratio survival analyses on the basis of our cohort data. Finally, we identified the lymphocyte component of NLR to be the primary driver of survival prognosis. To our knowledge, this is the largest US cohort utilized to analyze immunologic ratio biomarker-associated outcomes and perform dichotomized analyses for the purpose of identifying the prognostic driver of the NLR in surgical PDAC patients.

Inflammation and the inflammatory response have been discussed extensively in the literature in relation to tumorigenesis, progression, and metastasis. Furthermore, links have been established between the inflammatory response and oncogenic signaling pathway interactions, tumor microenvironment analyses, and use of immune-targeted therapies [22]. Surrogate biomarkers of inflammation have proven useful in predicting disease progression, recurrence, and overall prognosis across a wide range of malignancies [10, 11, 23–25]. In a meta-analysis evaluating the role of the systemic immune-inflammation index, Zhong et al. showed that an elevated systemic immune-inflammation index is associated with worse OS in hepatocellular carcinoma, urinary cancers, gastrointestinal cancers, and small-cell lung cancer [11]. In a review of 116 patients with gastrointestinal malignancies, Nora et al. demonstrated NLR and PLR to be significant predictors of lymph node positivity, metastatic disease, and recurrence, especially when used in combination [25]. The use of the NLR, PLR, and LMR have shown promise in pancreatic adenocarcinoma, demonstrating prognostic value in both resectable and palliative populations [17, 26].

The NLR has shown substantial potential for prognostic utility in pancreatic adenocarcinoma patients. In a large retrospective analysis of surgical PDAC patients, a low NLR (< 5) was associated with longer median survival (26 vs 13 months, *P* = .001), and an NLR ≥ 5 independently predicted poor prognosis (HR, 1.66 [95% CI 1.12–2.46]; *P* = .012) [15]. Giakoustidis et al. further explored pretreatment NLR in surgical PDAC patients and identified decreased OS rates to be associated with a high NLR in univariate analyses, which maintained independent prognostic significance in multivariable analyses [13]. Two recent meta-analyses including a total of 9771 patients have also suggested an association between NLR and OS, in which elevated NLR carried poor prognoses. Zhou et al. found elevated NLR to be associated with shorter rates of OS (HR, 1.81 [95% CI, 1.59–2.05]; *P* < .00001) and disease-free survival (HR, 1.66 [95% CI, 1.17–2.35]; *P* = .005) [27]. Evaluating OS alone, Mowbray et al. also demonstrated that significantly shorter rates of OS were associated with elevated NLR (HR, 1.77 [95% CI, 1.45–2.15]; *P* < .01) [28]. We corroborated these results in our own resected PDAC patients and similarly demonstrated that decreased rates of OS were associated with an NLR ≥ 5 in multivariable analyses. Additionally, we showed a significant association between high preoperative NLR and a decrease in RFS. Our study further supports the NLR as a valid prognostic biomarker for early-stage PDAC.

Although a cutpoint of 5 has been widely used to define high/low NLR, variations in cutpoints exists, with some groups using values ranging from 2 to 5 [15, 27–35]. With no clearly defined cutpoint, we chose to perform a continuous analysis to identify an optimal cutpoint for the NLR in relation to survival. Based solely on the data from our cohort, optimal cutpoints of 4.8 for OS and 4.9 for RFS were obtained. Our study supports the prognostic value of the commonly used NLR cutpoint of 5. As the NLR was the only significant ratio in our cohort, we elucidated its prognostic driver by analyzing the components of the ratio. The denominator, the lymphocyte count percentage, alone yielded a survival curve identical to the NLR, whereas the numerator, the isolated neutrophil count percentage, was not statistically significant, suggesting that lymphocyte count percentages have equal prognostic value and, perhaps, offer a simpler alternative to the NLR biomarker. This finding is supported by those from previous studies that showed low lymphocyte counts to be poor prognostic indicators in pancreatic and colorectal cancers [36–39]. The finding also has immunotherapeutic implications, which corroborate basic science findings on a population level [40–42]. 

In contrast to our study, other studies have found no prognostic significance of the NLR in some PDAC patient populations. Recently, Chawla et al. described a cohort of 217 resectable PDAC patients whose NLR at diagnosis did not correspond to OS [43]. Jamieson et al. similarly reported 135 patients who underwent PDAC resection and found no relationship between NLR and survival [29]. Similar findings have been reported by other groups [30, 31]. The reasons for this variability include diverse patient populations, differences in ratio cutpoints, timing of blood collections, and receipt of neoadjuvant therapy. In the current study, 37% of patients received neoadjuvant therapy before pancreatic resection, which may have influenced immune cell populations.

Increased monocyte presence in the tumor microenvironment or in circulation has been implicated in angiogenesis, tumor growth, and poor prognosis in cancer patients [44]. Circulating monocytes are commonly quantified by the LMR, which has demonstrated an inverse association with survival and prognosis in solid tumor malignancies [45]. Few studies have investigated this parameter in surgical PDAC patients. In a large review and meta-analysis of 1795 patients, Li et al. reported a favorable prognosis associated with elevated LMR in pooled analyses (HR, 0.56 [95% CI, 0.38–0.83]; *P* = .004) [16]. Although this study included a range of LMR cutpoints and both resected and nonoperable PDAC patients, a prognostic value of the LMR was observed in surgical patients in subgroup analyses [16]. Sierzega et al. reported a series of 442 resectable PDAC patients demonstrating prolonged median survival (29.2 vs 13.1 months, *P* = .001) in the LMR ≥ 3 group [15]. An LMR < 3 was an independent predictor of poor prognosis (HR, 1.65 [95% CI, 1.06–2.58]; *P* = .026) [15]. In contrast to studies previously discussed, Abe et al. demonstrated no association between LMR and OS or disease-free survival in a large retrospective analysis of the prognostic effects of patient-specific nutritional and immunologic factors in resected PDAC patients [17]. We also did not show a prognostic value of LMR in our analyses of resected PDAC patients. Differences in prognostic outcomes were likely due to the paucity of data evaluating LMR and survival, inconsistency in evaluated patient cohorts, and variation of cutpoint delineation. We used mean values for LMR cutpoints in our analyses because of the variation of cutpoints reported in the literature. An optimal cutpoint analysis of LMR for OS and RFS was performed to clarify the reporting of LMR associated outcomes.

Survival outcomes have similarly been linked to elevated PLR in solid tumor malignancies [46]. Compared to other commonly described ratios, the application of PLR to PDAC is less clear, with mixed outcomes reported. Giakoustidis et al. also investigated pretreatment PLR in surgical PDAC patients and identified decreased OS with high PLR in univariate analyses [13]. The PLR did not maintain independent prognostic significance in multivariable analysis. Interestingly, patients with concurrently high NLR and PLR experienced significantly decreased OS when compared to those with normal NLR and PLR or those with an elevation of either ratio (7, 48, 32%, respectively; *P* = .001) [13]. In a subsequent analysis of resected and inoperable PDAC patients, Stotz et al. found no association between OS (HR, 1.13 [95% CI: 0.82–1.57]; *P* = .46) and PLR (HR, 1.07 [95% CI, 0.82–1.40]; *P* = .61) in either cohort [33]. Similarly, no demonstrable association between PLR and OS was observed in several separate resected PDAC patient series [15, 29, 31, 35]. Consistent with the literature discussed above, our study did not find a significant correlation between survival (OS or RFS) and PLR in resected PDAC patients.

However, some authors have demonstrated the PLR to be an important predictor of survival. Smith et al. and Watanabe et al. reported elevated PLRs as the most significant determinant of survival in their resected PDAC cohorts of 110 and 46 patients, respectively [30, 47]. Reasons for inconsistent results may have included differing PLR cutpoint values, small patient cohorts, and variations in multidisciplinary treatments of these patients with complex PDAC. Furthermore, the PLR was synthesized using surrogates that are fundamental to many biologic functions (ie, coagulation cascade), which may explain the variability of correlation in oncologic outcomes. In our study, mean values were initially used for PLR cutpoints because of the variation reported in the literature. Again, an optimal PLR cutpoint analysis was performed to provide clarity and consistency in the reporting of PLR-associated factors.

The limitations of this study include those inherent in reviewing retrospective data. Although our data set was robust and associated with an electronic medical record, the potential for selection bias exists. Additionally, although all blood specimens were collected in the preoperative setting, there is potential for variation regarding the date and time blood draws were done in relation to the surgery date. The present study did not stratify patients based on receipt of neoadjuvant therapy. This stratification was previously investigated by our group, who reported significantly decreased rates of OS among patients with increased NLR after neoadjuvant therapy when compared to those with stable NLR [14]. Finally, we did not analyze pretreatment immunologic ratios in patients who received neoadjuvant chemotherapy; therefore, we were not able to determine whether chemotherapy significantly altered preoperative values.

## Conclusion

There continues to be little doubt about the importance of inflammation and immunity in cancer biology. The NLR and other immunologic ratios are derived from easily obtainable standard laboratory values, with little added expense. When obtained in the preoperative setting, the NLR is a biomarker with the potential to guide treatment algorithms in early-stage PDAC patients and provide clarity on common unresolved management dilemmas routinely debated today. Given their demonstrable poor outcomes, patients with high NLR may benefit from neoadjuvant systemic therapy variation, more detailed preoperative staging, or stratification in clinical trials. Additionally, consistent with the findings of developing research on the tumor microenvironment and immunotherapy, lymphocytes alone may be significant drivers of survival. In the context of improving outcomes, our results suggest targeting inflammatory pathways may be relevant in chemoprevention. Prospective trials would serve to elucidate the provided prognostic information and provide insight into alternative treatment algorithms that can improve outcomes among patients with PDAC.

## Supplementary information

**Additional file 1.** Summary statistics of immunologic ratios.

**Additional file 2.** Kaplan-Meier plot demonstrating overall survival (OS) in dichotomized NLR values: (a) Neutrophil and lymphocyte (b) percentage.

## Data Availability

The data that support the findings of this study are available from the corresponding author upon reasonable request.

## Abbreviations

CCI: Charlson Comorbidity Index
LMR: Lymphocyte to monocyte ratio
NLR: Neutrophil to lymphocyte ratio
OS: Overall survival
PDAC: Pancreatic ductal adenocarcinoma
PLR: Platelet to lymphocyte ratio
R0: Margin negative resection
RFS: Recurrence-free survival
