# Supplementary material for: Neutrophil to lymphocyte ratio, not platelet to lymphocyte or lymphocyte to monocyte ratio, is predictive of patient survival after resection of early-stage pancreatic ductal adenocarcinoma
Source: BMC Cancer. 2020 Aug 11;20:750. doi: 10.1186/s12885-020-07182-9 (PMC7422564; doi:10.1186/s12885-020-07182-9)
Supplement: Supplementary file 1 — Additional file 1. Summary statistics of immunologic ratios. [file 12885_2020_7182_MOESM1_ESM.docx]

| **Additional File 1.** Summary statistics of immunologic ratios | | | | | | | |
| --- | --- | --- | --- | --- | --- | --- | --- |
| **Ratio** | **Minimum** | **Maximum** | **Median** | **Mean** | **SD** | **Cut point** | **Population (N = 277) with given cut point, no. (%)** |
| **NLR** | 0.5 | 15.8 | 2.9 | 3.4 | 2.2 | < 5 | 239 (86.3) |
|  |  |  |  |  |  | ≥ 5 | 38 (13.7) |
| **PLR** | 56 | 816.7 | 144.4 | 169.1 | 95.7 | < 144.4 | 139 (50.2) |
|  |  |  |  |  |  | ≥ 144.4 | 138 (49.8) |
| **LMR** | 0.5 | 18.0 | 2.9 | 3.1 | 1.9 | ≤ 2.9 | 140 (50.5) |
|  |  |  |  |  |  | > 2.9 | 137 (49.5) |
| **Abbreviations**: NLR, neutrophil to lymphocyte ratio; PLR, platelet to lymphocyte ratio; LMR, lymphocyte to monocyte ratio; SD, standard deviation | | | | | | | |
