# Supplementary figures and images for: Neutrophil to lymphocyte ratio, not platelet to lymphocyte or lymphocyte to monocyte ratio, is predictive of patient survival after resection of early-stage pancreatic ductal adenocarcinoma
Source: BMC Cancer. 2020 Aug 11;20:750. doi: 10.1186/s12885-020-07182-9 (PMC7422564; doi:10.1186/s12885-020-07182-9)

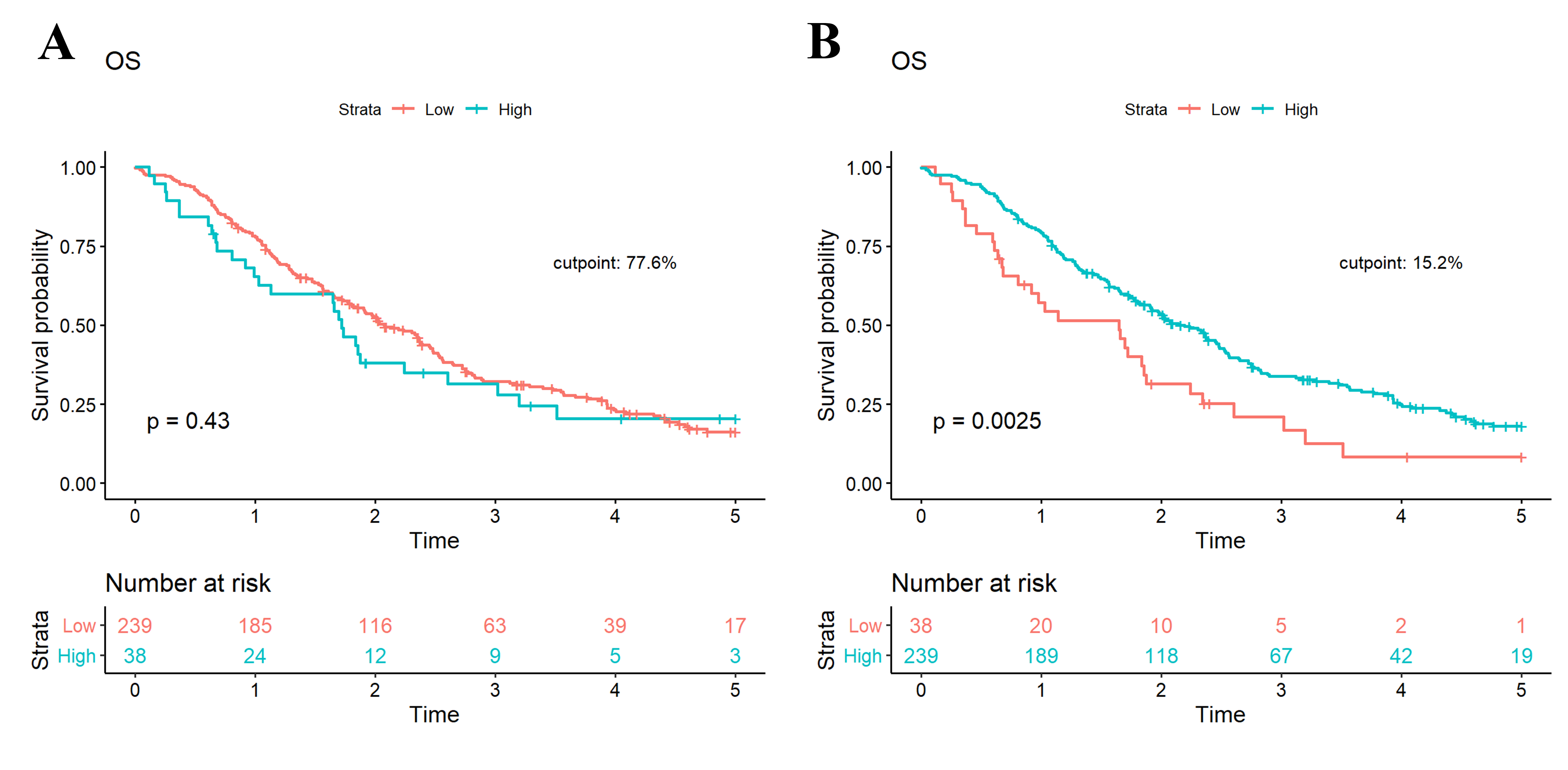

Supplement: Supplementary file 2 — Additional file 2. Kaplan-Meier plot demonstrating overall survival (OS) in dichotomized NLR values: (a) Neutrophil and lymphocyte (b) percentage. [file 12885_2020_7182_MOESM2_ESM.tif]
